# Alpha-synuclein pathology in the “weaver” mouse, a genetic model of dopaminergic denervation

**DOI:** 10.17912/micropub.biology.001156

**Published:** 2024-03-13

**Authors:** Aggeliki Dimopoulou, Vasiliki Panagiotakopoulou, Theodora Mourtzi, Ilias Kazanis, Fevronia Angelatou

**Affiliations:** 1 Department of Physiology, School of Medicine, University of Patras, Patras, 26500, Greece; 2 Laboratory of Human and Animal Physiology, Department of Biology, University of Patras, Patras, 26500, Greece; 3 current: Department of Cellular Neurology, Hertie Institute for Clinical Brain Research, University of Tübingen & German Center for Neurodegenerative Diseases (DZNE), Tübingen, D-72076, Tübingen, Germany; 4 Lab of Developmental Biology, Department of Biology, University of Patras, Patras, 26500, Greece; 5 School of Life Sciences, University of Westminster, W1W 6UW, London, UK

## Abstract

Alpha-synuclein plays a pivotal role in Parkinson’s disease (PD) pathogenesis, with α-synuclein aggregates/oligomers being identified as toxic species and phosphorylation at Serine 129 promoting aggregation/oligomerization. We investigated the biochemical profile of α-synuclein in the “weaver” mouse, a genetic PD model. Our results revealed increased Serine 129 phosphorylation in the midbrain, striatum, and cortex at a phase of established dopaminergic degeneration on postnatal day 100. These results indicate α-synuclein pathology already at this stage and the potential for age-related progress. Our findings confirm that the “weaver” mouse is an invaluable genetic model to study α-synuclein pathogenesis during PD progression.

**Figure 1. Biochemical profile of α-synuclein (α-Syn) in the Midbrain (MB), Striatum (STR), Cortex (CX), and histopathological stage of Substrantia nigra (SN) in “weaver” (wv/wv) and wild type (+/+) animals on postnatal day 100:**
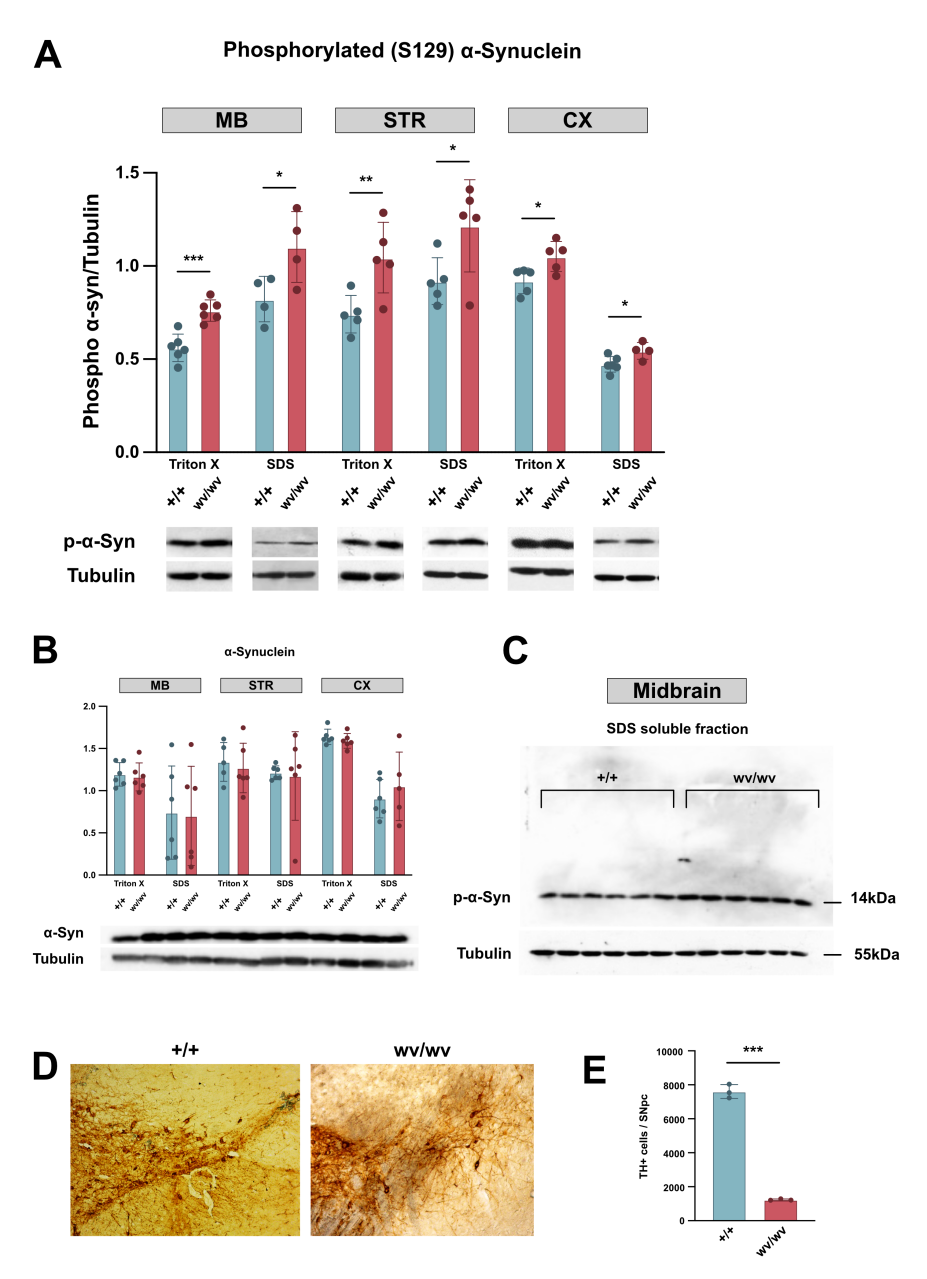
A) Scatter plot graph presenting the phosphorylation state of α-synuclein at Serine 129 (S129) as measured by western blot, using the phospho α-syn antibody. A significant increase of phosphorylated (S129) α-synuclein (p-α-Syn) levels was observed in wv/wv compared to +/+ animals, in both Triton X and SDS soluble fractions, across all three brain areas. The phosphorylated α-synuclein monomer (14 kDa) and Tubulin (55 kDa), serving as a loading control, are shown in cropped blots. [bars show averages and error bars standard deviations. * p<0.05, **p<0.01, ***p<0.001; using student’s t-test analysis] B) Scatter plot graph presenting the levels of total α-synuclein as measured by western blot, assessed using the Syn1 antibody. No difference in α-synuclein levels was detected between wv/wv and +/+ animals in either brain area, in either fraction. [bars show averages and error bars standard deviations. Statistical analysis was performed with student’s t-test analysis] C) SDS soluble fraction of MB of wv/wv and +/+ animals. The phosphorylated (S129) α-synuclein monomer (14kDa) can be seen in the cropped gel/blot. No species of higher molecular weight could be detected. D) Representative images of SN in “weaver” mice P100 (right) and age-matched wild-type mice (left). E) Corresponding quantification of TH+ immunolabelled neurons in SN of wild-type (+/+) P100 and “weaver” (wv/wv) animals. [bars show averages and error bars standard deviations. Statistical analysis was performed with student’s t-test analysis, *** p<0.001].

## Description

Parkinson’s disease (PD) is a prevalent neurological movement disorder, affecting more than 1% of the population by the age of 65 years (Poewe et al., 2017) . The hallmark neuropathological feature of PD is the progressive degeneration of dopaminergic neurons in the substantia nigra pars compacta (SNpc), leading to severe depletion of striatal dopamine. This process coincides with the appearance of intracellular proteinaceous inclusions termed Lewy bodies (LBs), containing aggregates of α-synuclein (Spillantini et al., 1997) . Alpha-synuclein oligomeric aggregates are well-characterized toxic species that play a significant role in the PD neurodegenerative process by disrupting various subcellular functions (Emmanouilidou et al., 2010; Colla et al., 2012; Choi et al., 2013) . Furthermore, phosphorylation of α-synuclein at Serine 129 has been shown to facilitate the accumulation of oligomeric α-synuclein *in vitro* (Anderson et al., 2006) and potentially regulate the onset of neuronal dysfunction *in vivo* by increasing the protein’s seeding capacity (Karampetsou et al., 2017) .

The “weaver” mouse model is the only genetic model of progressive nigrostriatal dopaminergic neurodegeneration (Triarhou et al., 1988; Ghetti and Triarhou, 1992) , initiating on postnatal day 7 (P7) and reaching 75% cell loss by P60 (Panagiotakopoulou et al., 2020) . It results from an autosomal recessive point mutation of the Girk2 (Kcnj6) gene, which encodes for a G-protein-activated, inwardly rectifying potassium channel (Patil et al., 1995) . The mutation leads to a loss of ion selectivity and induces dopaminergic neurodegeneration due to excitotoxicity (Slesinger et al., 1996) . Although Girk2 mutations have not been identified in human cases of PD, homozygous “weaver” mice exhibit numerous significant disease hallmarks. These include a reduction of dopamine levels in the striatum (Schmidt et al., 1982; Panagiotakopoulou et al., 2020) , motor deficits (Schmidt et al., 1982; Derenne et al., 2007; Panagiotakopoulou et al., 2020) , neuroinflammation (Peng et al., 2006) , reduced levels of brain-derived neurotrophic factor (BDNF) (Botsakis et al., 2017; Panagiotakopoulou et al., 2020) , and cognitive impairment in the more advanced stages of degeneration (Derenne et al., 2007) .

Recent evidence from our research group has shown that the midbrain of the “weaver” mouse exhibits significantly high levels of oxidative stress (Patsoukis et al., 2005; Botsakis et al., 2017 **).** Free, protein-bound, and total malondialdehyde (MDA) levels, which constitute key markers of lipid peroxidation, were measured, using a previously described method (Grintzalis et al., 2013) . Total MDA levels were increased in the midbrain of “weaver” mice by 241%, compared to wild-type animals on postnatal day 21 (P21), pointing out profoundly high levels of oxidative stress (Botsakis et al., 2017) . In addition, robust neuroinflammation in the midbrain of the “weaver” mice has been observed, as indicated by the high levels of inducible nitric oxide synthase (iNOS), a marker of inflammation associated with glial cells, on P21, as well as significantly activated microglia on P60 (Botsakis et al., 2017; Panagiotakopoulou et al., 2020) .

Several lines of evidence suggest that neuronal oxidative stress promotes the formation of α-synuclein aggregates/oligomers, contributing to the neurodegenerative process in PD (Puspita et al., 2017; Scudamore et al., 2018; Won et al., 2022) . Therefore, it is of great interest to investigate whether oxidative stress impacts α-synuclein in the “weaver” brain during the neurodegeneration process, thus rendering this mouse model an additional valuable tool to study the role of α-synuclein in PD.

To investigate the biochemical profile of α-synuclein, we performed sequential protein extraction (fractionation) on the midbrain (MB), the striatum (STR), and the cortex (CX) of “weaver” and wild-type animals on postnatal day 100 (P100), approximately two months following the assessment of oxidative stress. The two resulting fractions (Triton X-100 and SDS), serve to separate the different α-synuclein species, as the Triton X-100 fraction includes the soluble α-synuclein, while the SDS fraction mainly contains the -insoluble in Triton X-100- oligomeric and aggregated forms of the protein. Specifically, the aspects examined included:

a) the phosphorylation of α-synuclein at Serine 129 (Ser129)

b) the presence of α-synuclein oligomers

c) the histopathological stage of the Substantia Nigra (SN) on P100.

Our results revealed a significant increase in the phosphorylation of α-synuclein at Ser129 in the Triton X-100 soluble fraction across all three examined brain areas of the "weaver" mouse (wv/wv), compared to the wild-type animals (+/+). Specifically, in the MB, we observed an increase of 35.6% (wv/wv, 0.76±0.057, n=6 vs. +/+, 0.561±0.074, n=6) ( Fig. 1A, left ). Similarly, in the STR, we noted a 40.8% increase (wv/wv, 1.045±0.189, n=5 vs. +/+, 0.742±0.101, n=5) (Fig 1A, middle). In the CX, a lower yet significant increase of 14.2% was observed (wv/wv, 1.051±0.081, n=5 vs. +/+, 0.92±0.069, n=5) ( Fig. 1A, right ).

Interestingly, a significant increase in the phosphorylation (Ser129) was observed in the SDS soluble fraction, which mainly contains aggregated/oligomeric forms of α-synuclein, across all three brain areas of the "weaver" mouse (wv/wv), compared to the wild-type animals (+/+). More specifically, in the MB an increase of 34.1% was observed (wv/wv, 1.102±0.19, n=4 vs. +/+, 0.822±0.122, n=4) ( Fig. 1A, left ), while in the STR a 32.3% increase was found (wv/wv, 1.215±0.221, n=5 vs. +/+, 0.919±0.112, n=5) (Fig 1A, middle). In the CX, a lower yet significant increase of 15.3% was noted **(** wv/wv, 0.545±0.045, n=4 vs. +/+, 0.472±0.041, n=5) ( Fig. 1A, right ). Remarkably, no difference in the total amount of α-synuclein (Syn-1) was detected between the wv/wv and +/+ animals in the same brain areas ( Fig. 1B ).

This analysis presents the first compelling evidence of α-synuclein pathology in a genetic model phenocopying PD, the “weaver” mouse. The elevated levels of phosphorylated (Ser129) α-synuclein seen in the “weaver” MB, STR, and CX, specifically in the SDS fraction, indicates an enrichment of phosphorylated (Ser129) Triton X- insoluble species in these tissues, suggesting phosphorylated α-synuclein pathology. *In vitro* experiments have shown that the phosphorylation of α-synuclein at Serine 129 can promote the accumulation of oligomeric α-synuclein (Anderson et al., 2006) and accelerate the formation of α-synuclein inclusions (Smith et al., 2005; Sugeno et al., 2008; Rocha et al., 2018) . Moreover, *in vivo* experiments revealed that intracerebral injection of phosphorylated (Ser129) α-synuclein pre-formed fibrils (P-PFF) leads to increased seeding and accumulation of the endogenous α-synuclein and exacerbated pathology in the cortex (Karampetsou et al., 2017) . As highlighted earlier, the prefibrillar forms of α-synuclein, referred to as oligomers, are the early and toxic species that contribute to the neurodegenerative processes in PD (Emmanouilidou et al., 2010; Colla et al., 2012; Choi et al., 2013; Roberts et al., 2015; Rocha et al., 2018) . Approximately 90% of the α-synuclein deposited in LBs in the human brain is phosphorylated at residue Ser129 (Fujiwara et al., 2002; Anderson et al., 2006) .

In our immunoblot analysis, the increased levels of the insoluble phosphorylated (Ser129) α-synuclein seen in the SDS fraction (Fig.1.C), suggests that in the brain of the weaver mice, at least at the age of P100, a phosphorylated α-synuclein accumulation takes place. Thus, the “weaver” mouse could be proved to be a useful model to study the mechanisms involved in the process of phosphorylated α-synuclein accumulation. No higher molecular weight species of the protein, which represent the oligomeric forms of α-synuclein, could be detected in the SDS fraction ( Fig. 1C ). This demonstrates that either they are not present in the brain tissue examined or cannot be detected by the antibodies used.

The histopathological examination of the midbrain tissue revealed that on P100, 83,8% (wv/wv, 1233±49, n=3 vs. +/+, 7603±409, n=3) of dopaminergic neurons are lost, confirming that degeneration continues when compared to P21 and P60, where a 56% and 75% cell loss as observed, respectively (Botsakis et al., 2017; Panagiotakopoulou et al., 2020) .

In conclusion, our results indicate that α-synuclein pathology in the “weaver” brain is already present at P100, and this might lead to subsequent oligomerization and aggregation of the protein, exacerbating the pathology. If this hypothesis holds true, we will possess a new tool -an invaluable genetic model of PD- to study the dysfunction of α-synuclein during the development of the disease, providing insights into the progression of human pathology.

## Methods

**Animals**

All experiments were performed with male and female homozygous “weaver” (A ^w-J^ /A-Kcnj6 ^wv^ /J) mice of the B6CBACa strain and age-matched B6CBACa wild-type animals. The breeding and handling of the animals complied with the European Communities Council Directive Guidelines (86/609/EEC) for the care and use of Laboratory animals (Simon and Ghetti, 1994) as implemented in Greece by the Presidential Decree 56/2013 and approved by the Prefectural Animal Care and Use Committee (No: EL 13BIO04) and the Animal Welfare and Ethical Review Committee of the University of Patras. Animals were housed in cages in a steady 12h light/dark cycle room and had free access to food and water. Homozygous “weaver” mice identification was performed on P14 and was based on a distinctive phenotype, including tremor, weakness, gait instability, and ataxia (Simon et al., 1994) . The heterozygous individuals (wv/+) used for the breeding, since male wv/wv are infertile, were identified through genotyping (PCR).

The PCR protocol included the following steps: 1. Pro-incubation (95°C, 15min), 2. Denaturation (95°C, 30s), 3. Annealling (54.5°C, 30s), 4. Extension (72°C, 1min) and, 5. Final step (72°C, 10min). The number of cycles was 27.

**Brain region dissection and protein extraction (fractionation)**

Both wv/wv and +/+ mice were sacrificed on P100. The brain areas of interest (MB, STR, CX) were dissected and kept at -80 ^o^ C until the fractionation procedure. The tissue was directly dissolved in 1% v/v Triton X-100 buffer (150 mM NaCl, 50 mM Tris pH 7.6, 2 mM EDTA, 1% Triton X-100) by sonication, and then centrifuged at 100,000 g for 60 minutes. After the centrifugation, the supernatant was removed for the Triton X-100 fraction and the pellet was kept for the SDS fraction.

In more detail, the supernatant was carefully removed, placed in a new tube, and centrifuged at 50,000 g for 30 minutes. The resultant supernatant was stored at -80 ^o^ C as the Triton X-100 soluble fraction. The pellet, derived from the centrifugation of 100,000g, was resuspended in PBS 1X and centrifuged at 50,000g for further purification. The supernatant was removed, and the washed pellet was subsequently dissolved in 1% SDS buffer (50 mM Tris, pH 8.0, 150 mM NaCl, 5 mM EDTA, 1% NP-40, 0.5% sodium deoxycholate, 1% w/v SDS) by sonication. The dissolved pellet was then centrifuged at 100,000 g for 40 minutes, and the supernatant, after carefully removed, was centrifuged at 50,000g for 30 minutes. The obtained supernatant was stored, as the SDS soluble fraction, at -80 ^o^ C. Protease and phosphatase inhibitors (Roche, Switzerland) were added to both buffers, according to manufacturers’ instructions.

**Western Blotting**

For the western blotting, wv/wv and +/+ mice (P100) were used (n=4-6). Both protein fractions, Triton X-100 and SDS soluble, were analyzed in all three brain regions (MB, STR, CX) for each mouse. Protein concentration was estimated by NanoDrop 2000 (Thermo Fisher Scientific) and the DC protein assay (Bio-Rad, USA). The samples containing 35ug of protein were electrophoretically resolved on SDS polyacrylamide gel (13%) and transferred to nitrocellulose membrane. Afterward, the membranes were incubated with a blocking buffer containing 5% w/v milk dissolved in Tris-buffered saline with 0.1% Tween 20 detergent (TBS-T) for 60 minutes and subsequently with the primary antibody overnight at 4 ^o^ C. Detection was performed using horseradish peroxidase-conjugated secondary antibodies and an ECL kit (Merck-Millipore Billerica, Massachusetts, USA). For the antibody dilutions, the above blocking buffer was used, except for the anti-phospho (S129) α-syn, which was diluted to buffer containing 5% w/v BSA dissolved in TBS-T. Molecular weight was determined by comparison with the Bluestar pre-stained protein molecular weight marker standard (MW03, Nippon Genetics). The scanned images of membranes and band intensities were calibrated and quantified using NIH-Fiji ImageJ software (version 1.52p).

**Immunohistochemistry**

Animals were killed with intracardial infusion of 4% paraformaldehyde, and brain tissue was postfixed in 4% paraformaldehyde at 4 ^o^ C overnight before being cryoprotected in 30% w/v sucrose and frozen. Brain sections were processed for immunostaining following previously described protocols (Botsakis et al., 2017) . The primary antibody was an anti-TH rabbit polyclonal antibody (1:100, AB152, Chemicon, Merck-Millipore Billerica, Massachusetts, USA). Secondary antibody (anti-rabbit IgG, 1:200), avidin-biotin-peroxidase complex (ABC kit), and DAB kit (all Vector Laboratories, Burlingame, California, USA) were used according to the manufacturer's protocol. The number of TH-positive cell bodies in the SN was determined by unbiased stereology according to the optical fractionator method (West, 1999) . Cell counting was conducted bilaterally in brain sections using a digital camera coupled to a Nikon microscope (Nikon Eclipse TE2000-U microscope with the Nikon camera Digital Sight DS-L1) (Nikon Instruments, Tokyo, Japan) displaying the structure on a video monitor. The sections were identified using the mouse atlas of Franklin and Paxinos (2013).

## Reagents

**Table:** 

**ANIMAL**	**STRAIN/GENETIC BACKGROUND**	**GENE WITH MUTATION**	**GENOTYPE**	**OBTAINED**
“weaver” mouse	B6CBACa A ^w-J^	Kcnj6(Girk2)	Kcnj6 ^wv^ / Kcnj6 ^wv^	The Jackson Laboratory
wild-type mouse	B6CBACa A ^w-J^		Kcnj6 ^+^ / Kcnj6 ^+^	The Jackson Laboratory

**Table:** 

**PRIMARY ANTIBODY**	**DESCRIPTION/ DILUTION**	**SOURCE**
Phospho (S129) α-syn	Rabbit polyclonal specific to α-synuclein phosphorylated at Ser129 (1:1,000)	Abcam, 59264
α-syn (Syn-1)	Mouse monoclonal specific to α-synuclein (1: 1,000)	BD Transductions, 610787
Tubulin	Mouse monoclonal specific to α-Tubulin (1:20,000)	Cell Signalling, 3873
TH	Rabbit polyclonal specific to Tyrosine Hydroxylase (1:100)	Merck, AB152
**SECONDARY ANTIBODY**	**DESCRIPTION/ DILUTION**	**SOURCE**
Rabbit IgG	Goat conjugated to HRP (1:20,000)	Cell Signaling, 7074
Mouse IgG Fc	Goat polyclonal conjugated to HRP (1:20,000)	Thermo Fisher Scientific, A16084

**Table:** 

**PRIMER**	**SEQUENCE**
Forward “weaver” mutation allele	GAGACAGAAACCACCATCA
Forward wild-type allele	GAGACAGAAACCACCATCG
Reverse (common)	CACGGACTGGATTAAGAGGAGAATAAT
